# Review of the antioxidant potential of flavonoids as a subgroup of polyphenols and partial substitute for synthetic antioxidants

**DOI:** 10.22038/AJP.2023.21774

**Published:** 2023

**Authors:** Seyyed Hossein Hassanpour, Alireza Doroudi

**Affiliations:** 1 *Department of Nuclear Pharmacy, School of Pharmacy, Tehran University of Medical Sciences, Tehran, Iran*; 2 *Department of Medicinal Chemistry, Faculty of Pharmacy, Ahvaz Jundishapur University of Medical Sciences, Ahvaz, Iran*

**Keywords:** Antioxidant, Flavonoids, Food preservation, Phytochemical, Polyphenol

## Abstract

**Objective:**

This review describes the antioxidant activity of flavonoids as a subgroup of polyphenols and a partial or entire substitute for synthetic antioxidants.

**Materials and Methods:**

All relevant databases were searched using the terms “Phytochemical”, “Polyphenol”, and “Flavonoid”.

**Results:**

The oxidative reaction caused by free radicals is a reason for food spoilage, which causes unpleasant odor, loss of taste, and damaged tissues. The common antioxidants employed in foods include butylated hydroxyanisole, butylated hydroxytoluene, propyl gallate, and tert-butyl hydroquinone. Despite their high efficiency and potency, synthetic antioxidants have adverse effects on the human body, such as causing mutation and carcinogenicity. A whole and a group of them known as polyphenols possess high antioxidant activity. These compounds are potential antioxidants due to their capabilities such as scavenging free radicals, donating hydrogen atoms, and chelating metal cations. The antioxidant mechanism of action of flavonoids is transferring hydrogen atom to free radicals. Accordingly, the more the flavonoid structure makes the hydrogen transfer faster and easier, the more the flavonoid’s antioxidant power will be. Therefore, the antioxidant activity of the flavonoids with hydroxyl groups in their structure is the highest among different flavonoids.

**Conclusion:**

In addition to health promotion and some disease prevention effects, various *in vitro* investigations have indicated that flavonoids possess high antioxidant activity that is comparable with synthetic antioxidants. However, to be commercially available, these compounds should be extracted from a low-price source with a high-performance method.

## Introduction

Most food products are made up of various chemical components that are easily oxidized. Lipids (i.e., fats, oils, and waxes) compared to chemical compounds that cause oxidation reactions in most food products, have the most significant potential to lose electrons in general. Lipid auto-oxidation in food products initiated by metal ions exposure, metalloprotein catalysis, light, heat, or ionizing radiation can deteriorate the color, flavor, texture, quality, and safety of food. Food fats are chemically made of triglycerides, and oxidation occurs at the unsaturated sites of the triglycerides, resulting in rancidity (Atta et al., 2017; Latifi et al., 2019). Free radicals are the most frequent oxidants in biological systems. Free radicals are molecules, ions, or atoms, with one or more unpaired electrons in valence shell or outer orbit. The odd number of electron(s) of a free radical makes it unstable, short lived and highly reactive; as result, it is capable of independent existence. In order to stabilize themselves, unpaired electrons in these free radicals seek and acquire electrons from other substances. When the first assault is paired to the odd electron, another free radical is produced in the process, resulting in a chain reaction. Free radicals cause considerable damage to the macromolecules in body organisms, including deoxyribonucleic acid (DNA), proteins, lipids, and carbohydrates. The damages caused by free radicals are also referred to as oxidative damages (Halliwell, 2012; Hassanpour and Karami, 2021; Nogala-Kalucka et al., 2005; Daneshniya et al., 2020). 

Free radicals are frequently obtained from nitrogen, oxygen, and sulfur molecules in biological systems. Such free radicals are the constituents of reactive sulfur species (RSS), reactive nitrogen species (RNS), and reactive oxygen species (ROS) molecular families. Free radicals, such as superoxide anion (O2-•), nitric oxide, hydroxyl radical, per-hydroxyl radical, and other species, such as hydrogen peroxide, singlet oxygen (O2), hypochlorous acid, and peroxynitrite (ONOO-), are forms of ROS. Nitric oxide is converted to RNS by reacting with O2-• to generate ONOO-. By the reaction of thiols with ROS, RSS is quickly produced. The ROS production occurs due to cellular metabolism and functional activities (Atta et al., 2017; Daneshniya, 2020). 

**Figure 1:**
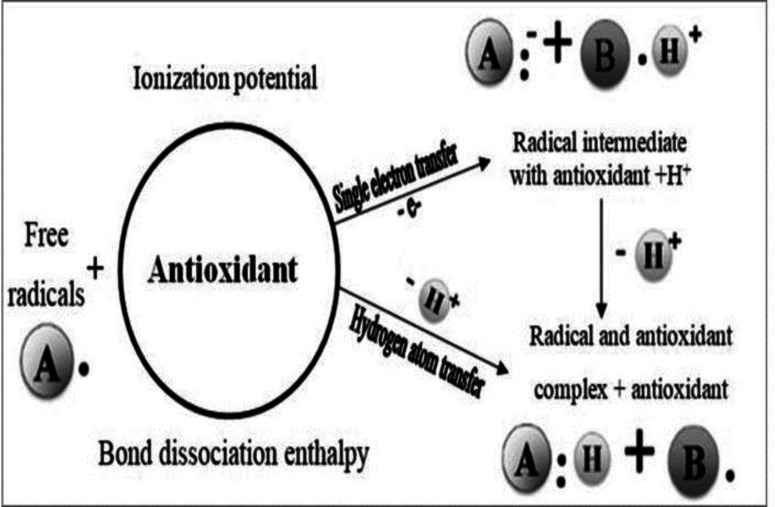
Schematic representation of common antioxidant mechanism

Antioxidants are among the factors that prevent oxidative damage. The structure of antioxidants is such that although they release hydrogen atoms, the reactivity of these atoms is extremely low (Pokorný, 2007). Figure 1 depicts the common schematic mechanism of antioxidants. In general, antioxidants are divided into synthetic and natural groups. Because synthetic antioxidants are cheaper and more accessible and show high efficiency and stability, they were gradually considered alternatives to their natural type after World War II (Halliwell et al., 1995; Afanas' ev et al., 1989). The nonnutritive part of plants (i.e., phytochemicals) has a positive effect on metabolism, cancer, nervous system, wounds, mouth, and teeth. One of the reasons for turning to phytochemicals is their antimicrobial properties; therefore, they are grown in laboratories for easy and extensive access. The adjustment of cultural conditions for propagation shows signs of the future success of this method in order to be used in medicine (Bansal and Priyadarsini, 2021).

Phytochemicals especially the phenolic compounds are important natural antioxidants and are recognized as excellent antioxidants due to their capacity to scavenge free radicals, donate electrons and hydrogen atoms, and chelate metal cations (Halliwell et al., 1995, Latifi et al., 2021). Currently, synthetic antioxidants are more commonly used in industries, the most common of which are butylated hydroxytoluene (BHT), tert-butyl hydroquinone (TBHQ), butylated hydroxyanisole (BHA), and propyl gallate (P.G.). Several other compounds have been suggested to have antioxidant activity; nevertheless, only a few can be used in foods. Ethoxyquin is a synthetic antioxidant with a nonphenolic structure, the use of which is not permitted for humans, compared to other previously mentioned antioxidants; however, it is used for food preservation in animal feeding (Latifi et al., 2019). 

Using antioxidants in foods has been limited by the regulatory laws of one's country or international standards (Dorman et al., 2004). As a result, the consumption of synthetic antioxidants is limited due to their toxic effects and carcinogenicity. Since the carcinogenicity of synthetic antioxidants has been observed, the necessity of using alternatives without adverse effects is becoming increasingly important (Ardabili et al., 2010). 

Natural antioxidants have shown strong antimicrobial activity and can be used as a potential alternative to chemical antioxidants. Additionally, many of these compounds are known by the United States Food and Drug Administration (FDA) as safe compounds that are used extensively in food compounds (Prakash B et al., 2020). Therefore, due to increasing consumer concern about synthetic antioxidants due to their toxic effects, the demand for natural antioxidants, especially of plant origin, has increased recently (Formanek et al., Biswas et al., 2004; Jayathilakan et al., 2007). This review study was conducted to investigate the antioxidant activity of phytochemicals, emphasizing on the capacity of flavonoids to be utilized as natural antioxidants in food and examine their capability to substitute for synthetic antioxidants.

## Materials and Methods

This review study was conducted to investigate the antioxidant activity of phytochemicals, especially flavonoids, and their potential to entirely or partially substitute for synthetic antioxidants in food products. All relevant databases were searched for the terms “Phytochemical”, “Polyphenol”, “Flavonoid”, “Natural Additive”, and “Antioxidant Activity”. 

The data were collected using PubMed, Scopus, Web of Science, Google Scholar, and ScienceDirect databases, related books, and prominent conferences and congresses with various publication dates ranging from 1981 to 2021.

## Results

 Phytochemicals are bioactive substances that are beneficial for human health. These compounds are categorized by their chemical structures and include polyphenols (i.e., flavonoids and nonflavonoid polyphenols), phytosterols, carotenoids, organosulfur, and betalains. Phytochemicals as natural antioxidants prevent the change in color, taste and odor of food, etc., which is caused by the oxidation process and play an important role in the plant defense system. More than 5000 pigments in plants are considered to be phytochemicals. These compounds can reveal their biological activity through various bioactivities, such as antiviral, antibacterial properties, antioxidant, anti-inflammatory, anticancer, modulation of enzyme activities, stimulation of the immune system, and regulation of cholesterol synthesis, gene expression, and blood pressure. 

Phytochemicals can also improve health and prevent diseases (Puri et al., 2012; Yadav and Agarwala, 2011). Due to the presence of these compounds in fruits and vegetable-rich diets, they can delay aging, reduce inflammation and the oxidative stress risk, and decrease the risk of chronic diseases, such as arteriosclerosis, cardiovascular diseases, cancers, cataracts, diabetes, neurological diseases, and cognitive function disorders (Eliassen et al., 2912; Pojer et al., 2013; Tanaka et al., 2012). Vegetables, fruits, legumes, and whole grains are the primary sources of phytochemicals in a diet (Tokusoglu Ö., 2011). There are different methods for extracting phytochemical compounds. These compounds can be extracted from plants through physical and chemical methods, such as ultrasonication, supercritical fluid extraction, solvent extraction, and cold pressing (Puri et al., 2012; Latifi et al., 2019). 

It should be pointed out that several phytochemicals are insoluble in aqueous solutions and do not have acceptable oil solubility. These compounds are chemically unstable and decompose promptly after exposure to the external environment due to temperature, oxygen, pH, light, and other reactive substances. Phenolic compounds or polyphenols are important groups of phytochemical compounds in plants (Bravo, 1998; Daneshniya et al., 2021). Phenolic compounds have excellent antioxidant characteristics and are often found in fruits and vegetables (Heim et al., 2002). These compounds include flavonoids, flavanols, anthocyanins, anthraquinones, and their benzoyl and acetylated compounds. 

Polyphenols are found in two general classes, namely flavonoids and phenolic acids. Different parts of a plant produce compounds that are mainly phenolic and are known as natural antioxidants. Several compounds, such as flavonoids, coumarins, carotenoids, tocopherols, organic acids, and derivatives of cinnamic acid, are phenolic antioxidants from the plant sources. In this category of compounds, the hydroxyl group is directly bonded to an aromatic hydrocarbon (Amorati and Valgimigli, 2012). 

**Figure 2:**
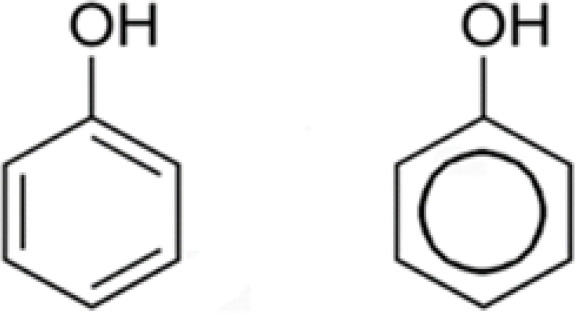
Phenol, the most straightforward component of phenolic compounds

There are at least 8000 structures of phenolic compounds known, the simplest of which is phenol, shown in Figure 2 (Bravo, 1998).

Phenolic compounds are known for their antioxidant activity that depends on the structure, especially the location and number of hydroxyl groups and the nature of substituents on aromatic rings (Balasundram et al., 2006). Phenolic compounds are referred to as health promotion compounds with the capability of preventing chronic heart disease (Harborne and Williams, 2000; Holtekjølen et al., 2008). The antioxidant characteristics of phenolic compounds depend on their capability of donating electrons to trap free radicals by the formation of stable phenoxyl compounds (Lam et al., 2007). 

Plants have always been excellent food sources for the consumption of valuable bioactive compounds (Tayel and EL‐TRAS, 2012). Such natural antioxidants are extracted from plants in the form of essential oils and extracts from various sources, such as fruits (e.g. pomegranates, grapes, dates, and kinnow), vegetables (e.g. potatoes, broccoli, drumstick, Indian turmeric, pumpkin, and nettle), and medicinal plants and spices (e.g. rosemary, tea, cinnamon, oregano, common sage, thyme, ginger, peppermint, and clove), and have been assessed for reducing fat oxidation (Devatkal et al., 2009; Huang et al., 2011; Wójciak et al., 2011; Das., 2012; Latifi., 2019; Ebadi et al., 2021). Phenolic compounds are discovered in combination with saccharides (monosaccharides and polysaccharides) bonded to one or more phenolic groups. Although some phenolic compounds are ubiquitous, others are specific to particular plant families and are found in particular plant organs or at specific stages of plant growth (Cheynier, 2012). Table 1 lists important phenolic compounds in plants that have been reported to possess antioxidant activity in *in vivo* and *in vitro* studies.

**Structures of flavonoids and their antioxidant activity**

**Figure 3:**
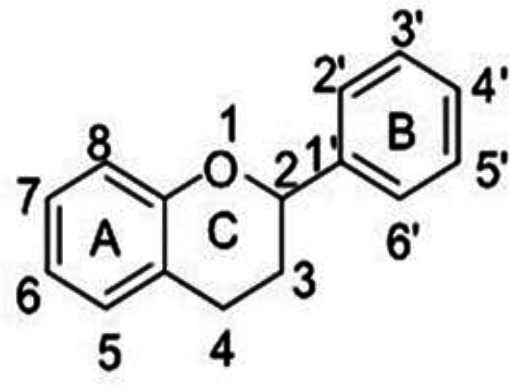
Basic structure of flavonoids (Phenylbenzopyrane)

Flavonoids are the largest group of phytochemicals and are considered to be the most widespread group of polyphenols available in vegetables and fruits. Flavonoids have excellent antioxidant properties and can exhibit their antioxidant activity by scavenging free radicals and ROS, chelating metals, and preventing the oxidation of low-density lipoproteins (LDLs) (Heim et al., 2002; Chu et al., 2000). Flavones are the most basic structures of flavonoids. From the structural point of view, all flavonoids possess a C6-C3-C6 carbon skeleton, where the carbon atoms are located in three phenolic A, B, and C rings, and the C ring normally contains oxygen (Pietta, 2000; Ververidiset al., 2007). Figure 3 depicts phenyl-benzopyrone as the basic structure of flavonoids. Flavonoids are found in different families, each of which has different members. The flavonoid families include flavones, isoflavones, flavonols, flavanones, dihydroflavonols, flavan-3-ols (monomers), proanthocyanidins (oligomeric flavan-3-ols), and anthocyanidins. Table 2 shows these families and their members (Laura et al., 2019). 

**Regulations regarding synthetic and natural antioxidants**

There is a need to move away from synthetic antioxidants due to an increasing interest in natural food antioxidants; however, the synthetic or natural origin of the different antioxidants that are applied in the food industry are unknown in the official tables indicating the levels and permissions to use each additive in each food type (Gutiérrez-del-Río et al., 2021). The Codex Alimentarius Commission is the worldwide authority in charge of regulating and establishing the framework of food regulations that guide the use of antioxidants in foods. Since food regulatory systems and legal frameworks regarding using antioxidants as food additives differ by country, these criteria are neither required nor immediately applicable (Manessis et al., 2021). 

**Table 1:** The phenolic compounds with high antioxidant activity

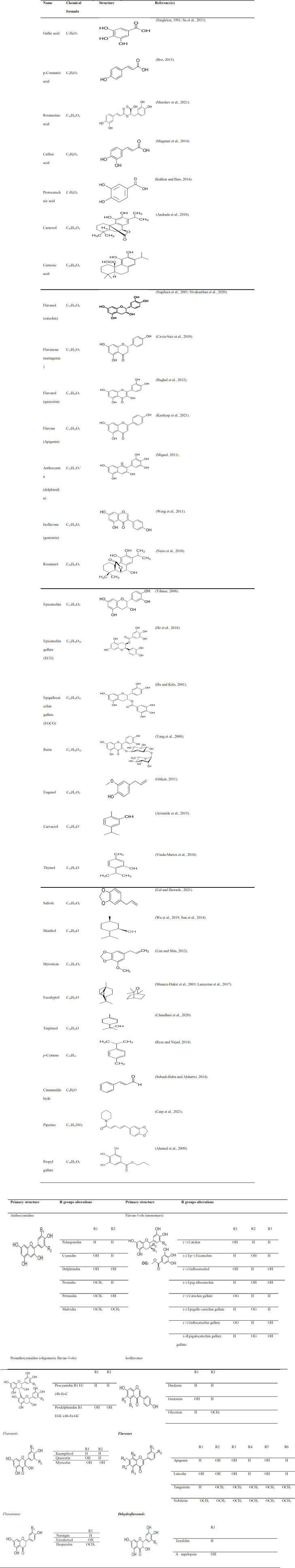

The European Food Safety Authority of the European Union (E.U.) and the United States FDA are the two primary guiding authorities in charge of regulating the licensing of food additives worldwide (Carocho et al., 2018). Under the E.U., regulation E.C. No 1129/2011 specifies some antioxidants, categorized based on their E-numbers, that are regulated in the “other food additives” section. The natural antioxidants recognized as food additives by the E.U. include rosemary extracts (E392), tocopherols (E306–E309), and ascorbic acid (E300), according to this regulation (European Parliament and Council Commission Regulation, 2011). 

Food products and ingredients are monitored by various authorities in the United States, the most relevant of which is the United States FDA. The FDA’s regulations for food items are listed in Title 21 of the Code of Federal Regulations of the United States. There is no distinct category for natural antioxidants in the Code of Federal Regulations, as there is in the European regulations (Oswell et al., 2018; FDA Title 21 Part 172, 2022; FDA Title 21 Part 182, 2021). Although natural antioxidants, such as tocopherols and ascorbic acid, are specifically designated for use in food, the United States regulation is far more comprehensive than the European one and includes additional substances that belong to other categories and still have demonstrated antioxidant activity. Several of the compounds allowed for use as spices, coloring adjuncts, or natural flavorings, such as flavonoids, phloretin glycoside, and carotenoids, such as astaxanthin and carotene, or extracts of sage or rosemary, have been recognized with the antioxidant potential. Astaxanthin, phloretin, and sage extract are not listed as food additives in any category in E.U. regulations, although they are classified for technical applications in United States standards (FDA Title 21 Parts 73, 2018; European Commission Food Additives, 2022). Considering the above-mentioned regulations, it is evident that regardless of the utilization of natural or synthetic antioxidants, the optimal choice of antioxidant for each food matrix should be determined on a case-by-case basis because *in vitro* antioxidant activities cannot always be generated in the food, and frequent changes occur due to food processing or interactions with other components of food matrix that have prooxidant or antioxidant activities (Barden and Decker, 2016). 

**Table 2:** Flavonoids family

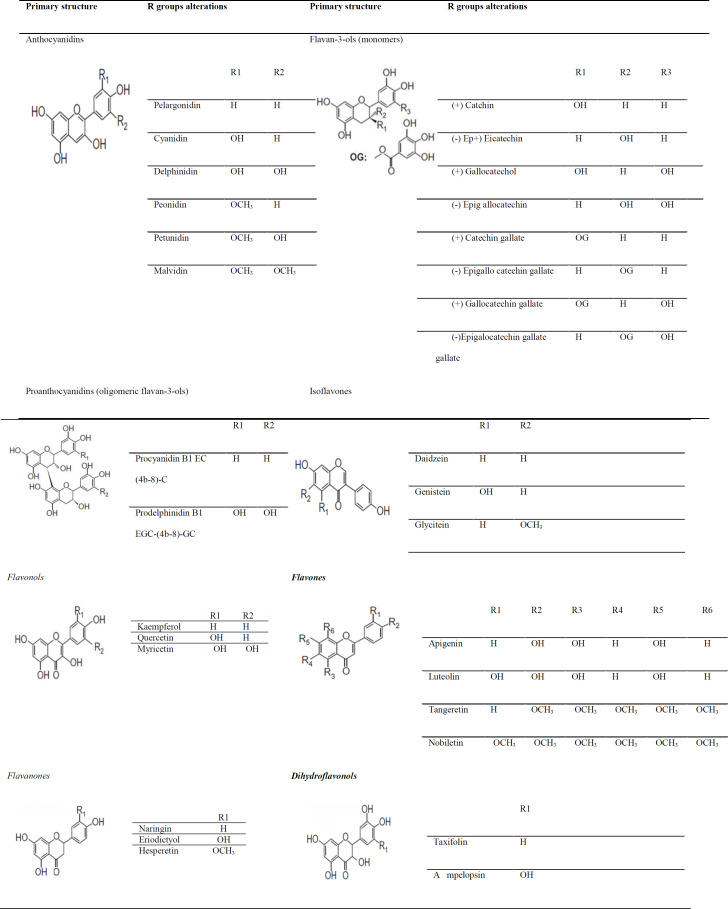

**Antioxidants in food preservation**

Once lipid oxidation in a food matrix is analyzed over time, there is frequently a lag phase, where the deposition of lipid oxidation by-products is moderate. Such lag phase can be associated with the existence of antioxidants in the food matrix, limiting the synthesis of free radicals targeting fatty acids and the less free radical generation that precedes the concentration of ß-scission reactions and hydroperoxides (Gutiérrez-del-Río, 2021). The objective of the food industry is to extend the lag period, where the concentrations of the components that cause rancidity taste are less than human detection limits. When the food matrix lacks natural antioxidants and/or includes significant endogenous prooxidants, the most typical technique used by food manufacturers to limit oxidation is adding antioxidants directly to the food matrix (Gutiérrez-del-Río, 2021). 

In the context of food science, the phrase “antioxidant” refers to substances that prevent lipid peroxidation (LPO) and other oxidative reactions, thereby preserving the shelf-life extension and freshness of foods. The action method is the same whether the antioxidant is synthetic or natural and comprises O_2 _quenching, metal chelating, and free radical scavenging (Yang et al., 2018; Lin et al., 2016). Due to their stability, low cost, and wide availability, synthetic antioxidants are frequently employed. Phenolic antioxidants are the most extensively applied synthetic antioxidants in the food industry, with BHT (E321), TBHQ (E319), P.G. (E310), and BHA (E320), as the most common ones. Table 3 shows some critical information regarding the chemical structures and health-related concerns about the consumption of these antioxidants (Kwan et al., 2014; Additives and Nutrient Sources, 2011; Additives and Nutrient Sources, 2012; Additives and Nutrient Sources, 2016; Additives and Nutrient Sources, 2014; Additives and Nutrient Sources, 2015; Additives and Nutrient Sources, 2016; Additives and Nutrient Sources, 2015; Additives and Nutrient Sources, 2016; Nutritional Dietetics Allergy Products, 2015). 

Despite the fact that such synthetic antioxidants are widely used and tightly regulated, their safety should be considered owing to overdose usage and/or misuse; for instance, combining various antioxidants might exacerbate their toxic effects. High concentrations of synthetic chemical antioxidants can induce *in vitro* toxicity or DNA damage in some tissues (Lourenço et al., 2019; Xu, b2021; Liu and Mabury, 2020; Baran et al., 2021). The food industry is attempting to reduce using synthetic compounds by substituting natural alternatives against consumer concerns that they are being exposed to potentially toxic synthetic chemical compounds through their regular diet. Although plant antioxidants are safe; but for more assurance; toxicity tests should always be performed for all cases (Lourenço et al., 2019).

**Structure of flavonoids and their antioxidant properties**

Flavonoids are bioactive polyphenol compounds that are found in almost all fruits and vegetables. Aglycones, glycosides, and methylated derivatives are different forms of flavonoids. Aglycones are produced when a hydrogen atom replaces the glycosyl group in a glycoside. Glycosides are composed of two parts, sugars (glycans) and nonsugars (aglycones), and play different roles in plants (Daneshniya et al., 2020). Various species of flavonoids differ in oxidation rate and C-ring substituents; in other words, the existence of one double bond, one carbonyl group, and one hydroxy group in the pyranyl C ring is used to classify them as species and subspecies. The substituents of A and B rings with hydroxy groups are used to identify the available members in a species. 

**Table 3:** Chemical structures of food industry antioxidants

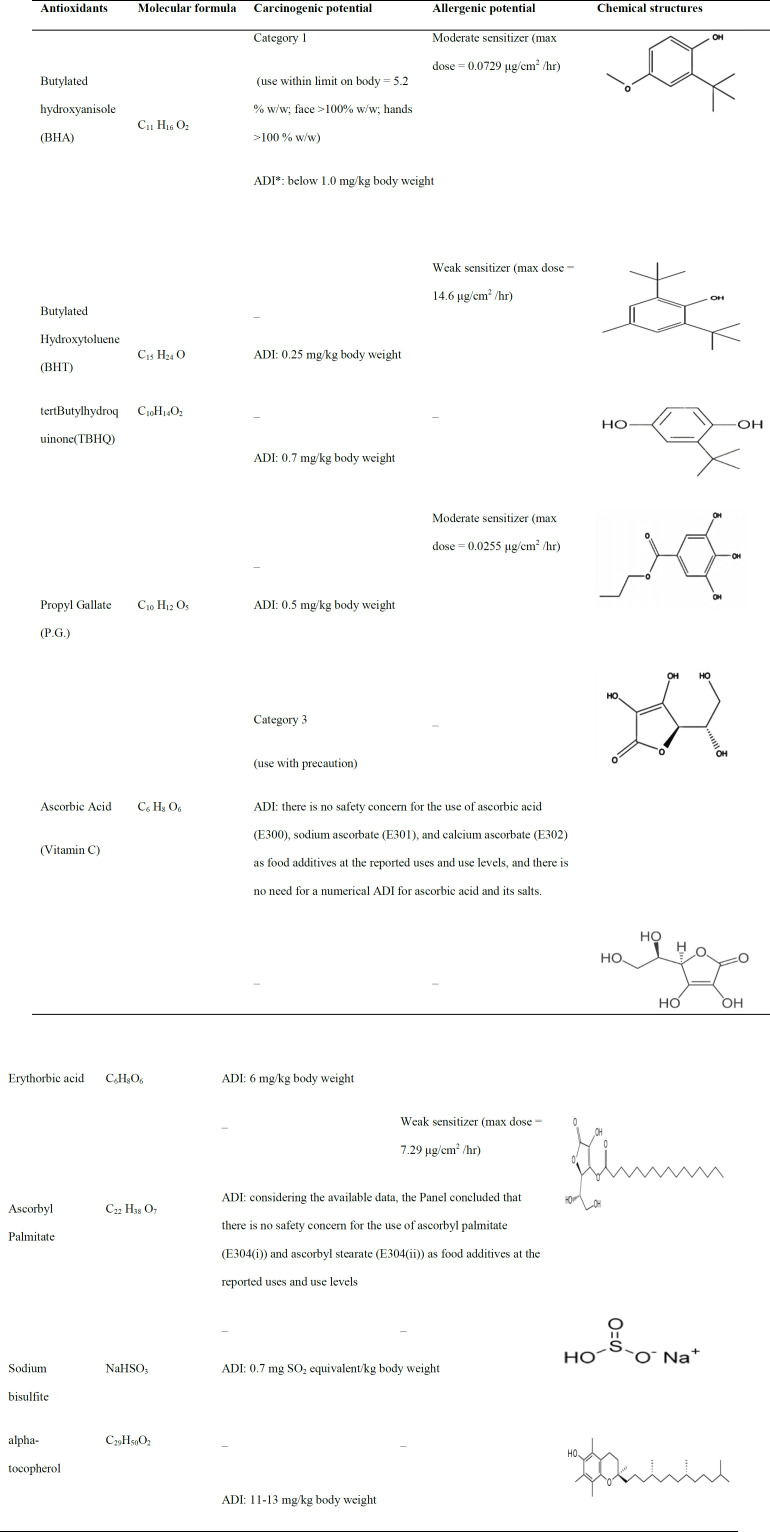

Flavonoids in plants are often derived from glycosylate and play a role in the production of shining blue, reddish-yellow, and reddish-orange shades in leaves, flowers, and fruits. Regardless of the variety of fruits and vegetables, flavonoids are found in seeds, nuts, buds, spices, herbal medications, some drinks, such as wine, especially red and tea wines, and in smaller quantities in beer. The antioxidant properties are among the most important properties of flavonoids. In numerous studies, flavonoid-rich plant extracts have been used to prevent food oxidation (Daneshniya et al., 2020). Despite the many benefits of synthetic antioxidants, compared to those of natural ones, such as their low price, using antioxidants in food has been limited by the regulatory laws of international standards or one country (Dorman et al., 2004). Therefore, the use of synthetic antioxidants is limited due to their toxic effects and carcinogenicity. Synthetic antioxidants have a limit of using 0.02% fat in food (Daneshniya et al., 2020). Since the carcinogenic properties of synthetic antioxidants have been observed, the necessity of the use of alternatives without adverse effects has become increasingly important (Ardabili et al., 2010). Therefore, in recent years, the demand for natural antioxidants, particularly of plant origin, has risen as a consequence of consumers’ concerns regarding such synthetic antioxidants due to their potential toxicological impacts (Jayathilakan et al., 2007). 

In general, flavonoids are antioxidants with high efficiency and potency, and as a result, by reducing the oxidation of LDL, they protect the body against cardiovascular diseases. Flavonoids both prevent LPO and act as scavengers of radicals, such as superoxides, lipid peroxides, and hydroxylated compounds, and lead to the inactivation of single oxygen molecules and prevention of the activity of lipoxygenases. The high potency of flavonoids in inhibiting free radicals relates to their ability to transfer a hydrogen atom from a hydroxyl group to the free radical and ultimately stabilize it as follows:

FLOH + R° → FLO° + R.H.

Flavones and catechins are the most powerful flavonoids with antioxidant properties that can protect the body against ROS. The free radical scavenging activity of flavonoids is as follows (Gülçin, 2012):

Myricetin>quercetin>rhamnetin>morin>diosmetin>naringenin>apigenin>catechin>5,7-Dihydroxy-3,3',4'-trimethoxyflavone> robinin > kaempferol > flavone

Different factors affect the antioxidant properties of flavonoids. One of the most important factors is the structure of flavonoids. The presence of hydroxyl and glycosylated groups has a significant effect on the antioxidant activity of flavonoids. The existence of glycosylated groups reduces the antioxidant properties of flavonoids. On the other hand, the presence of hydroxy groups increases the antioxidant properties of flavonoids. For maximum free radical scavenging activity, flavonoids should have a hydroxy group at the 4' and 3' positions of the B ring, a conjugated double bond between carbon 2 and 3, an oxo group at the 4 positions of the C ring, a hydroxy group on carbon 3 in the C ring, and a hydroxy group on carbon 5 in the A ring (Figure 3). The presence of many hydroxy groups, especially in the B ring, leads to an increase in the antioxidant activity of flavonoids. The hydroxyls of the B ring are the first active stations in traversing the oxidation chain. The three structural groups are responsible for determining the scavenging activity of free radicals and antioxidant activity of flavonoids, including a catechol section in the B ring, a conjugated double bond at the positions 2 and 3, an oxo group with the carbonyl group function in the ring C, and the existence of hydroxy groups at the positions 3 and 5 (Gülçin, 2012; Heijnen et al., 2001). Therefore, it is noteworthy that flavonoids can exhibit different antioxidant activities according to their structure.

Flavonoids are classified as nonnutrients in the scientific food realm. They have typically been eliminated from food crops due to their inhibitory effects on digestive enzymes, astringency and bitterness, and poor absorption after intake. However, due to their inclusion in regular meals, several therapeutic benefits, such as antioxidant properties in animal studies, the decrease of cardiovascular disease and high blood pressure, and antiallergic, anti-inflammatory, and anti-diabetic activities, were recognized. As a result, flavonoids are now considered third-order functional components (food factors) that have biological regulatory roles (Terahara, 2015). 

Flavonoids are also important in food preservation and industry. The phrase “sustainable intensification” was created by the Royal Society of London, United Kingdom, which denotes current agricultural practices aiming at raising food production while also safeguarding biodiversity and environmental processes. Food waste minimization is one of the most critical elements of “sustainable intensification”. As a result, there is a growing demand for postharvest storage technology and food preservatives research (D'Amelia et al., 2018; Petersen and Snapp, 2015). 

Since today’s consumers are concerned about using chemically synthesized preservatives, natural products are prioritized. At the same time, industries are giving a greater focus on using plant-derived antioxidant and antibacterial components to improve shelf life. The antioxidant effect of flavonoids might preserve food stability over time and offer protection against necrotrophic fungi and food-borne diseases. The flavonoids’ antioxidant capacity in food systems is associated with their capacity to prevent lipid autoxidation as a primary cause of food quality degradation and shelf-life reduction (Shahidi and Ambigaipalan, 2015). Flavonoids are capable of donating hydrogen atoms to lipid radicals, resulting in more stable antioxidant radicals that are less susceptible to autoxidation. The antioxidant action mechanisms of flavonoids include direct ROS scavenging, inhibition of ROS generation via the chelation of trace elements (quercetin has iron-stabilizing and iron-chelating effects), or inhibition of free radical-generating enzymes (i.e., microsomal monooxygenase, glutathione S-transferase, nicotinamide adenine dinucleotide phosphate oxidase, and mitochondrial succinoxidase), and activation of antioxidant defenses (i.e., the up-regulation of antioxidant enzymes characterized by radical scavenging capacity). A synergy of some of such mechanisms, such as radical scavenging activity along with some enzymes’ function inhibition, can also occur (Kumar and Pandey, 2013; Agrawal, 2011; Kaleem and Ahmad, 2018). 

The capacity of flavonoids to lower the susceptibility of fresh vegetables and fruits to particular postharvest infections might also contribute to their antioxidant effect in extending shelf life (Neha and Pandey-Rai, 2014). Flavonoids, exceptionally high hydroxylated anthocyanins, can prevent the growth of grey mold (produced by Botrytis cinerea), causing the dynamics of the ROS burst to be disrupted during infection. Flavonoids might inactivate and bind proteins and can form complexes that have bacterial cell walls, giving them antibacterial action against numerous pathogens (Zhang et al., 2013; Zhang et al., 2015; Hintz et al., 2015). 

Flavonoids have been employed in a variety of dietary applications due to their beneficial characteristics. Flavonoids have been applied as active antioxidant agents in the oxygen-sensitive food packaging industries to extend shelf life and improve the bioactive component levels (López de Dicastillo et al., 2011). Flavonoids have also been applied to minimize lipid oxidation in cooked pork patties and raw mackerel fillets (Viji et al., 2015). Considering all the above-mentioned information regarding the antioxidant potential of flavonoids, it is evident that, in addition to their health-promoting effects, they can be utilized as food additives to extend shelf life.

## Discussion

As one of the significant subgroups of polyphenols, flavonoids are crucial secondary chemicals known as phytochemicals generated by plants that are involved in various activities, including growth and development and stress resistance. The high demand and interest in using flavonoids in food processing and their health-promoting effects have resulted in the increased acknowledgment of the positive aspects of flavonoids for human health. It is evident that as an important subset of the phytochemical’s family, flavonoids possess a considerable antioxidant activity that is competitive with synthetic antioxidants. Flavonoids are abundant in vegetables, flowers, and seeds, and approaches have been developed for extracting these compounds from these natural sources for use as food preservatives and additives. However, on a commercial scale, the extraction of flavonoids directly from the aforementioned sources does not seem to be profitable. Therefore, it seems that attempting to find a good source for extraction, such as plant wastes, and designing a high-performance extraction method would be critical steps for these compounds to be applied in food products as preservatives.

## Conflicts of interest

The authors have declared that there is no conflict of interest.
